# Effect of dietary sesame (*Sesame indicum* L) seed meal level supplemented with lysine and phytase on performance traits and antioxidant status of late-phase laying hens

**DOI:** 10.5713/ajas.19.0107

**Published:** 2019-04-15

**Authors:** Payam Baghban-Kanani, Babak Hosseintabar-Ghasemabad, Saba Azimi-Youvalari, Alireza Seidavi, Vito Laudadio, Domenico Mazzei, Vincenzo Tufarelli

**Affiliations:** 1 Department of Animal Science, University of Tabriz, Tabriz 51666-14766, Iran; 2 Department of Animal Science, Urmia University, Urmia 57561-51818, Iran; 3 Department of Animal Science, Rasht Branch, Islamic Azad University, Rasht 41335-3516, Iran; 4 Department of DETO, Section of Veterinary Science and Animal Production, University of Study of Bari ‘Aldo Moro’, Valenzano 70010, Bari, Italy

**Keywords:** Hens, Egg, Atherogenic Index, Antioxidant Capacity, Superoxide Dismutase, Yolk Cholesterol

## Abstract

**Objective:**

This study was performed to investigate the effects of supplementing sesame seed meal (SSM) with phytase and lysine on performance, egg quality, blood biochemical and antioxidant status of laying hens.

**Methods:**

A total of 960, 56-wk-old laying hens were divided into 12 dietary groups with eight replicates per group (10 birds per replicate). A completely randomized design with factorial arrangement 2×3×2 consisted of two levels of lysine supplement (0% and 10% over requirement), three SSM levels (0%, 10%, and 20%) with or without phytase (0 and 300 g/ton). The feeding trial lasted 10 weeks.

**Results:**

Birds fed diets with 10% SSM had higher feed intake than groups fed 0% and 20% SSM. The addition of phytase to experimental feeds, improved feed conversion ratio, increased egg weight and mass (p<0.01). Egg quality criteria was not affected by supplementing phytase; however, supplementing 300 g/ton phytase to hens diet, led to a significant (p<0.05) increase in egg shell strength. Egg yolk cholesterol and serum low-density lipoprotein cholesterol, atherogenic index and total cholesterol were decreased (p<0.01) by diet containing 20% SSM. The high-density lipoprotein cholesterol was increased (p<0.05) in serum of hens fed 20% SSM than the other groups. It was also observed that total antioxidant capacity and total superoxide dismutase content of hens fed 20% SSM was significantly higher than control group (p<0.05).

**Conclusion:**

As from results, dietary supplementation of SSM and phytase had no negative effects on laying hens performance or egg quality while improving the egg oxidative stability.

## INTRODUCTION

The main constraint racing the poultry industry is the feed cost, which approximately represents 75% of the total cost production. Soybean meal (SBM) is the main protein source in poultry rations given its high nutritional value, and most of the Asian countries, including Iran, import this feed ingredient. Therefore, cottonseed, rapeseed, sunflower and sesame meals have been suggested as alternative protein sources for poultry diet [1].

Sesame (*Sesame indicum* L) seed is one of the most important oil crops and is composed of 45% to 50% lipid, 15% to 20% protein, and 10% to 15% carbohydrates [2]. Because of sesame seed meal (SSM) is the residue after oil extraction from seeds, it is an excellent protein source [3]. Sesame seeds have large variation in their composition due to the differences in cultivar, soil, cultural practices, climate and storage conditions. Research conducted on SSM indicated that it may provide an acceptable alternative to SBM in layer rations when substitution level is 50% or less [4]. However, Abdalla [5] reported that SSM has a lysine content of 2.7% (−50% compared to SBM) concluding that SSM can be used in limited amounts in poultry diet due the low lysine content. On the contrary, SSM is a valuable source of methionine (3.2%), high in fiber and low in metabolizable energy, unless processing procedures are used to remove the seed hull from meal. Moreover, SSM has high amounts of phytic acid which is well known to reduce calcium and phosphorus availability, and therefore it is recommended to add calcium when using SSM in poultry ration. The positive effects of phytase in laying hens are documented [6–8] and microbial phytase supplementation has been shown to increase crude protein and amino acids digestibility [1]. The reason for the use of phytase enzyme in the present trial was due to the presence of high amounts of phytic acid in SSM.

Therefore, the objective of this study was to investigate the effects of the dietary supplementation of SSM with phytase and lysine on performance, egg quality parameters, blood biochemical features and antioxidant status of laying hens.

## MATERIALS AND METHODS

### Animals and diets

The experimental protocol was approved by the Animal Ethic Committee of the Islamic Azad University, and the experiment conducted in respect to the International Guidelines for Research involving animals (Directive No. 2010/63/EU). A total of 960, 56-week-old Hy-Line (W-36) laying hens were assigned to 12 dietary treatments with eight replicates having 10 hens, in a 2×3×2 factorial arrangement by completely randomized design. The hens were allocated in individual cages (41×23×43 cm) and four cages were considered as one replicate. Before of starting the experiment, egg production of hens was measured individually and hens with similar egg production were replaced in each replicate. Diets were formulated using UFFDA software [9]. Dietary treatments consisted of two levels of lysine supplement (0% and 10% over requirements), three SSM levels (0%, 10%, and 20%; the SSM was included as partial substitute of SBM, wheat and corn) with or without phytase (0 and 300 g/ton). The SSM was provided from a commercial company (Arya, Urmia, Iran). The phytase, that used in this study, was NatuphosV–Phytase (Vetaque Company, Tehran, Iran), a dry stabilized preparation from fungal (*Aspergillus niger*) origin. Phytase was added at the levels of 0 and 300 g/ton, with a minimum guaranteed phytase level of 1,000 FTU/g. One FTU is the quantity of enzyme that releases 1 μmol of inorganic P/min from 0.00015 mol/L sodium phytate at pH 5.5 at 37°C. Birds were fed on a balanced commercial layer diet covering their daily requirement for two weeks prior to start the study to allow them to adapt and reach a standard level of egg production (data not shown). The chemical composition of SSM was determined according to AOAC [10], as reported in Table 1. The apparent metabolisable energy corrected to zero N-retention (AMEn) of the SSM was estimated according to the equation proposed by the World’s Poultry Science Association [11] as: AMEn (kcal/kg dry matter) = 16.23 (crude protein)+11.65 (crude fiber)+ 3.811 (nitrogen-free extract). The ingredient and nutritional composition of the diets are reported in Table 2. Feed and water were offered *ad libitum* during the whole experiment. Light was provided for 16 h per day during the trial period. The feeding trial lasted 10 weeks.

### Sample collection

During the experiment, daily feed consumption, egg weight, egg mass, egg production, feed conversion ratio (FCR) and mortality were measured. Body weight of hens was recorded at the beginning and at the end of the trial. Feed intake was measured weekly by subtracting the left-over feed from the quantity supplied to birds. Eggs from individual hens were daily collected and weighed. The egg production and feed efficiency were calculated as rate of production per hen per day and feed intake/egg mass. Egg quality parameters (shell thickness, shell strength, yolk index, Haugh unit, and yolk color) were assessed 24 h after egg collection. Shell strength was measured by a specific instrument (Digital Egg Shell Force Gauge, Wagner Instruments, Greenwich, CT, USA). Shell thickness was measured at three locations (air cell, equator and sharp end) using a digital micrometer (0.01 mm; Mitutoyo, Kawasaki, Japan). The yolk height (HY) was measured by tripod micrometer (0.01 mm; Mitutoyo, Japan) and the yolk diameter (D) by a compass (0.02 mm; Swordfish, Tokyo, Japan). The yolk index was calculated by the formula (Yolk index = [HY/D]×100). Haugh unit was calculated with following formula where the HA is albumen height and WE is egg weight (Haugh unit =100 log HA+7.57−1.7 WE^0.37^). Egg yolk colour was scored using the 15-point scale (colour scale from 15, dark orange to 1, light pale) of the DSM yolk color fan (DSM Nutritional Products Ltd., Basel, Switzerland). At the end of the experiment, 240 eggs from each treatment were collected for determination of yolk cholesterol. One gram of each egg yolk was homogenized with 15 mL of chloroform-methanol 2:1 (by volume), sonicated and filtered as previously described [1]. At the end of the experiment, blood samples (n = 6) per replicate were taken from wing vena into additive free blood tubes. Serum was obtained following centrifugation at 4,000 *g*×10 min at 20°C. Serum was separated to determine antioxidant capacity, concentrations of malondialdehyde (MDA), triglyceride, cholesterol, low-density lipoprotein cholesterol (LDL-C) and high-density lipoprotein cholesterol (HDL-C), using a commercial diagnostic kits (enzyme method) [1]. Then the obtained samples were stored at −20°C until further analysis. The atherogenic index was calculated as the ratio of LDL cholesterol to HDL cholesterol (LDL/HDL).

### Antioxidant status

Antioxidant capacity, including total antioxidant capacity (T-AOC), total superoxide dismutase (TSOD) and glutathione peroxidase (GSH-Px) was determined in serum samples using RANDOX kits (Wülfrath, Germany) according to the manufacturer’s instruction. Serum TSOD activity was assayed by the xanthine oxidase method [12], which monitors the degree of inhibition of nitroblue tetrazolium reduction by O_2_-generated by xanthine and xanthine oxidase; the absorbance was read at 550 nm using a spectrophotometer (UV-1201, Shimadzu, Kyoto, Japan). Serum lipid peroxidation (LP) was determined using the method proposed by Kei [13] and Yagi [14], but with 1,1,3,3-tetraethoxypropane as the standard. This method is based on the reaction between MDA (an aldehyde LP product) and thiobarbituric acid (TBA). The MDA forms a pink-colored complex with TBA. The absorbance of solution containing the complex was measured at 532 nm using a spectrophotometer (UV-1201, Shimadzu, Japan). The serum LP values were expressed in terms of MDA as nmol/mL plasma.

### Statistical analysis

Data were subjected to one-way analysis of variance (ANOVA) with 12 treatments and eight replicates having 10 hens in each replicate. Data obtained were submitted to ANOVA using the general linear model procedure of SAS software [15]. Means were compared by the Duncan’s multiple range test at 5% probability.

## RESULTS

The effects of experimental diets on performance and egg parameters of laying hens are shown in Table 3. The levels of SSM in diet influenced significantly feed consumption of layer hens (p<0.05), where birds receiving diet with 10% SSM had higher feed intake than the groups fed 0% and 20% SSM. Supplementing phytase led to a decrease in FCR (p<0.05) and increased egg weight and mass (p<0.01). The effects of the dietary treatments on egg quality parameters of laying hens are summarized in Table 4. Egg shell strength was significantly increased by phytase supplementation (p<0.05). Yolk weight and cholesterol of laying hen are shown in Table 5, where it was shown that the dietary treatments had no effect (p>0.05) on yolk weight, but egg yolk cholesterol (as mg/g of yolk) was significantly decreased (p<0.05) by diet containing 20% SSM. The Table 6 shows the effect of experimental diets on serum biochemical parameters of laying hens. In our study, triglycerides were not affected by dietary treatments (p>0.05). Including 20% SSM decreased (p<0.05) serum LDL-C, atherogenic index and total cholesterol (p<0.01). The serum HDL-C was significantly increased (p<0.05) hens fed 20% SSM than the control group. The effect of dietary treatments on serum GSH-Px activity, MDA, TSOD, and T-AOC is shown in Table 7. There were no significant effects of on serum GSH-Px and MDA, whereas T-AOC and TSOD contents of hens fed 20% SSM were significantly higher (p<0.05) than control group.

## DISCUSSION

The findings of the present study indicate that dietary supplementation with 20% SSM decreased feed consumption of laying hens. In contrast to this study, Cheva-Isarakul and Tangtaweewipat [4] and Mamputu and Buhr [16] reported that substituting SBM with SSM had no significant effect on feed intake in layers. According to our results, Al-Harthi and El-Deek [2] showed that broilers fed diets containing SSM consumed less feed as compared to unsupplemented diet. Our findings are also in line with those recently published by Sina et al [17] and Rezaeipour et al [3] reporting that Japanese quails decrease feed intake when fed dietary sesame meal. The reduction of feed intake with higher dietary levels of SSM in present experiment might be due to its content of bitter substances.

Phytase addition to diets affected on egg weight, egg mass and FCR, which is consistent with the results reported by Um and Paik [18] who reported that enzyme supplementation to diet increased hen-day egg production by 2.15% and egg weight by 0.29%, respectively. On the other hand, Boling et al [19] found that egg mass, egg production, egg weight, body weight, and feed intake of hens were not significantly improved by adding microbial phytase (300 U/kg) to corn-SBM diets with 0.15% available phosphorus. Ravindran [20] suggested that difference in bird performance fed diets with phytase supplement may be due to a number of factors including phytase source, feed ingredients, and dietary characteristics. The significant effect of phytase supplementation on FCR in the present study is in agreement with those of Liebert et al [21], concluding that enzyme supplementation to the corn-SBM diet improves FCR in laying hens. Recently, Kim et al [22] suggested that beneficial effects of phytase has been associated with more liberated available phosphorus from phytate-phosphorus, which can decrease its anti-nutritional effect and can generate myoinositol showing vitamin like or lipotropic effects. This increased utilization of phytate-phosphorus may further improve the utilization of energy and other nutrients such as amino acids and minerals in diets, which is known as an extra-phosphoric effect of phytase [23]. In our study, addition of phytase in diet significantly increased egg shell strength, and this may be due to increase calcium and phosphorus availability in phytase supplemented-diet [24]. These results are somewhat different from the results of Um and Paik [18], who reported that the egg shell strength from hens fed diet supplemented with enzyme was not significantly influenced. It may be speculated that increasing dietary phytase levels, phosphorus and calcium concentrations in diet were inadequate to support the proper eggshell formation in the current experiment. The egg yolk cholesterol decreased significantly by supplementation of 20% SSM in the layer diet. Kurtoglu et al [25] stated that in most animals, cholesterol is eliminated by catabolism and excretion in feces as biliary acids, but hens eliminate considerable amounts of cholesterol in the egg. Furthermore, there is a positive correlation between egg cholesterol and serum cholesterol. Also, the fiber content of SSM (as in Table 1) may stimulate binding of cholesterol with bile acids, and the inhibition of micelle formation combined with the effect of fermentation on short chain fatty acids production, mechanisms that have been proposed to explain the potential cholesterol lowering effects [1,26,27]. Cholesterol metabolism in laying hens has been studied by determining the effect of the dietary factors on the level of blood and egg yolk cholesterol. In the current experiment, SSM at the level of 20% decreased LDL-C, atherogenic index and total cholesterol, and also increased HDL-C in serum. In agreement with our findings, Alipoor et al [28] reported that sesame lignans (sesamin and/or episesamin) reduced serum total cholesterol and LDL-C concentrations by inhibiting the absorption and synthesis of cholesterol. In rat, the mechanism for the hypocholesterolemic effect of sesamin is believed to be related to inhibition of intestinal absorption of cholesterol, increased excretion of cholesterol into bile and decreased activity of 3-hydroxy-3-methylglutaryl coenzyme-A reductase [28]. In the literature review, we did not find any trial on the effects of SSM on HDL-C and atherogenic index in laying hens. So, direct comparisons cannot be easily made. We found that serum T-AOC and TSOD activity values increased by adding SSM. The antioxidant effects of SSM may cause this situation. It has been reported that sesamolin, a sesame seed lignan, reduced LP in rats [28]. Sesamin and sesamolin may enhance the effect of vitamin E and reduce LP as antioxidants [29]. Enzymatic antioxidants, such as TSOD and GSH-Px, play an important role in the conversion of reactive oxygen species to oxygen and water. The TSOD is a well-known scavenger enzyme preventing the cell from oxidative stress. Cells maintain their vital functions against oxidative damage with the help of a system that involves GSH-Px, SOD, catalase, glutathione reductase, some trace elements, and vitamins A and E. The increase of TSOD and T-AOC may be due to decreased utilization, since LP levels are low. Vitamin E has been recognized as one of the body’s major natural antioxidants; and sesame oil contains about 40 mg of vitamin E per 100 g oil [28]. Sesamin also might play a role in antioxidation by inhibiting the catabolism of tocopherol, resulting in enhanced accumulation of tocopherols in serum and tissues [30].

In conclusion, considering the laying hens feed consumption and FCR, the most suitable SSM level in diet was 10% having also no negative effects on performance or egg quality. Further, the present findings indicated that phytase addition improved hens performance and egg quality traits. Egg yolk cholesterol level could be reduced up to 5% by supplementing 20% SSM. Therefore, based on our results, SSM at 20% is of interest as a potential egg cholesterol-lowering agent, which would be helpful for the marketing of eggs and egg products. The addition of 20% SSM to the laying hen diet led also to an increase in serum TSOD activity and T-AOC amount. At the same time, the oxidative stability of egg was improved by SSM supplementation, and this may favorably influence the shelf life of eggs.

## Figures and Tables

**Table 1:** Chemical composition and metabolizable energy content of sesame seed meal

Nutrients	%
Dry matter	90.20
Crude protein	28.40
Ether extract	8.52
Crude fiber	15.11
Ash	9.30
NFE	38.67
AME_n_ (kcal/kg)	1,874
Ca	1.37
P	1.09
PP	0.74

NFE, nitrogen-free extracts; AME_n_, apparent metabolisable energy corrected to zero N-retention; P, phosphorus; PP, phytic phosphorus.

**Table 2:** Ingredient and chemical composition of experimental diets

Items	Lys 0.72%			Lys 0.82%		
	
	01)	10	20	0	10	20
Ingredients (%)						
Corn	49.00	47.64	46.25	49.04	47.71	46.32
Wheat	20.00	20.00	20.00	20.00	20.00	20.00
Soybean meal (44% CP)	18.70	12.51	6.20	18.40	12.19	5.88
Sesame meal (28% CP)	-	10.00	20.00	-	10.00	20.00
Vegetable oil	1.00	1.00	1.00	1.00	1.00	1.00
Oyster shell	9.99	7.93	5.58	10.00	8.03	5.70
Dicalcium phosphate	0.32	-	-	0.41	0.01	-
Vitamin premix2)	0.25	0.25	0.25	0.25	0.25	0.25
Mineral premix3)	0.25	0.25	0.25	0.25	0.25	0.25
DL-Meth (98%)	0.12	-	-	0.12	-	-
Salt	0.30	0.30	0.30	0.32	0.30	0.30
HCL- Lys	0.04	0.09	0.14	0.18	0.23	0.29
Phytase	0.03	0.03	0.03	0.03	0.03	0.03
Chemical composition (%)						
AME_n_ (kcal/kg)	2,795	2,795	2,795	2,795	2,795	2,795
Crude protein	14.32	14.32	14.32	14.32	14.32	14.32
Crude fiber	3.01	4.03	5.07	2.96	4.01	5.05
Calcium	4.51	4.51	4.51	4.51	4.51	4.51
Available P	0.30	0.30	0.36	0.31	0.30	0.35
Meth	0.32	0.34	0.39	0.35	0.34	0.38
Meth+Cys	0.54	0.61	0.67	0.61	0.61	0.67
Lys	0.72	0.72	0.72	0.82	0.82	0.82

CP, crude protein; AME_n_, apparent metabolisable energy corrected to zero N-retention.

1) Levels of sesame meal included.

2) Vitamin supplement provides per kg/diet: vitamin A, 8,000 IU; vitamin E, 20 IU; menadione, 3.0 mg; vitamin D_3_, 2,000 IU; riboflavin, 4.0 mg; pantothenate, 12 mg; nicotinic acid, 50 mg; choline 300 mg; vitamin B_12_, 15 mg; vitamin B_6_, 0.12 mg; thiamine, 1.5 mg; folic acid, 1.00 mg; d-biotin, 0.10 mg.

3) Mineral supplement provides as mg/kg diet: Mn, 100; Zn, 70; Fe 50; Cu 10; Iodine 1; Se, 0.30; antioxidant 50.

**Table 3:** Effect of experimental diets on performance and egg parameters of laying hens

Items	Feed consumption (g/d/bird)	Egg production (%)	Egg weight (g)	Egg mass (g/d/bird)	FCR
Lys (%)					
0.72	103.95	83.47	63.35	52.88	1.96
0.82	103.93	83.49	63.45	52.98	1.96
SEM	0.19	0.18	0.19	0.20	0.008
SSM (%)					
0	103.94^ab^	83.46	63.40	52.92	1.96
10	104.36^a^	83.70	63.64	53.28	1.95
20	103.52^b^	83.28	63.14	52.60	1.97
SEM	0.23	0.22	0.23	0.25	0.01
Phy (g/ton)					
0	103.86	83.31	63.07^b^	52.55^b^	1.97^a^
300	104.02	83.65	63.72^a^	53.31^a^	1.95^b^
SEM	0.19	0.18	0.19	0.20	0.008
Lys×SSM×Phy					
0.72×0×0	103.82	83.42	63.24	52.75	1.96
0.72×10×0	104.37	83.67	63.42	53.07	1.96
0.72×20×0	103.34	82.89	62.66	51.95	1.99
0.82×0×0	104.05	83.35	63.20	52.68	1.97
0.82×10×0	104.32	83.38	63.23	52.72	1.98
0.82×20×0	103.27	83.17	62.71	52.15	1.98
0.72×0×300	103.81	83.53	63.64	53.15	1.95
0.72×10×300	104.52	83.71	63.69	53.32	1.96
0.72×20×300	103.83	83.64	63.42	53.04	1.95
0.82×0×300	104.10	83.53	63.54	53.08	1.96
0.82×10×300	104.23	84.05	64.24	53.99	1.93
0.82×20×300	103.63	83.44	63.79	53.24	1.94
SEM	0.47	0.44	0.47	0.50	0.02
p-value					
Lys	0.96	0.96	0.70	0.74	0.76
SSM	0.05	0.41	0.33	0.17	0.78
Phy	0.57	0.19	0.01	0.01	0.03
Lys×SSM	0.78	0.99	0.90	0.92	0.76
Lys×Phy	0.85	0.87	0.54	0.56	0.57
SSM×Phy	0.79	0.84	0.71	0.63	0.81
Lys×SSM×Phy	0.97	0.68	0.83	0.71	0.70

FCR, feed conversion ratio; SEM, standard error of the mean; SSM, sesame seed meal.

a,b Means within a column with no superscript do not differ significantly (p>0.05).

**Table 4:** Effect of experimental diets on egg quality of laying hens

Items	Shell thickness (mm)	Eggshell strength (kg/cm^2^)	Shape index (%)	Haugh unit	Egg specific gravity (g/cm^3^)
Lys (%)					
0.72	0.32	3.44	74.19	81.79	1.13
0.82	0.32	3.45	74.17	82.21	1.13
SEM	0.001	0.01	0.32	0.25	0.008
SSM (%)					
0	0.31	3.42	74.17	81.92	1.13
10	0.32	3.44	74.19	81.95	1.13
20	0.32	3.47	74.19	82.12	1.13
SEM	0.002	0.01	0.39	0.31	0.01
Phy (g/ton)					
0	0.32	3.43^b^	74.18	81.78	1.13
300	0.32	3.47^a^	74.19	82.21	1.13
SEM	0.001	0.01	0.32	0.25	0.008
Lys×SSM×Phy					
0.72×0×0	0.31	3.42	74.16	81.75	1.14
0.72×10×0	0.32	3.42	74.16	81.60	1.13
0.72×20×0	0.32	3.46	74.22	81.59	1.14
0.82×0×0	0.31	3.44	74.16	81.77	1.13
0.82×10×0	0.32	3.43	74.17	81.93	1.13
0.82×20×0	0.32	3.44	74.20	82.03	1.13
0.72×0×300	0.31	3.43	74.17	81.84	1.13
0.72×10×300	0.32	3.45	74.21	81.99	1.13
0.72×20×300	0.32	3.48	74.25	81.99	1.14
0.82×0×300	0.32	3.44	74.19	82.34	1.13
0.82×10×300	0.32	3.45	74.23	81.29	1.14
0.82×20×300	0.32	3.50	74.09	82.87	1.14
SEM	0.004	0.03	0.7.9	0.63	0.02
p-value					
Lys	0.82	0.71	0.96	0.26	1.00
SSM	0.51	0.12	0.99	0.89	0.96
Phy	0.50	0.05	0.98	0.23	0.92
Lys×SSM	0.82	0.89	0.99	0.88	0.85
Lys×Phy	0.71	0.30	0.97	0.68	0.86
SSM×Phy	0.93	0.89	0.99	0.94	0.98
Lys×SSM×Phy	0.97	0.97	0.99	0.95	0.99

SEM, standard error of the mean; SSM, sesame seed meal.

a,b Means within a column with no superscript do not differ significantly (p>0.05).

**Table 5:** The effects of experimental diets on yolk weight and yolk cholesterol

Items	Yolk weight (g)	Yolk cholesterol (mg/yolk)	Yolk cholesterol (mg/g of yolk)
Lys (%)			
0.72	13.35	145.31	10.87
0.82	13.31	146.43	11.00
SEM	0.18	2.89	0.14
SSM (%)			
0	13.29	150.22	11.30^a^
10	13.37	145.27	10.86^ab^
20	13.32	142.13	10.65^b^
SEM	0.22	3.53	0.18
Phy (g/ton)			
0	13.26	143.88	10.84
300	13.39	147.87	11.03
SEM	0.18	2.89	0.14
Lys×SSM×Phy			
0.72×0×0	13.12	145.14	11.08
0.72×10×0	13.44	143.28	10.66
0.72×20×0	13.31	140.48	10.51
0.82×0×0	13.22	150.23	11.35
0.82×10×0	13.25	143.67	10.48
0.82×20×0	13.24	140.15	10.50
0.72×0×300	13.47	152.18	11.31
0.72×10×300	13.37	146.05	10.91
0.72×20×300	13.39	144.45	10.74
0.82×0×300	13.37	153.34	11.46
0.82×10×300	13.42	148.08	11.02
0.82×20×300	13.34	143.14	10.74
SEM	0.45	7.07	0.36
p-value			
Lys	0.87	0.78	0.54
SSM	0.97	0.27	0.03
Phy	0.61	0.33	0.34
Lys×SSM	0.99	0.91	0.93
Lys×Phy	0.97	0.90	0.82
SSM×Phy	0.94	0.98	0.99
Lys×SSM×Phy	0.94	0.96	0.99

SEM, standard error of the mean; SSM, sesame seed meal.

a,b Means within a column with same or no superscript do not differ significantly (p>0.05).

**Table 6:** Effect of experimental diets on serum biochemical parameters of laying hens

Items	LDL cholesterol (mg/dL)	HDL cholesterol (mg/dL)	Atherogenic index	Triglycerides (mg/dL)	Total cholesterol (mg/dL)
Lys (%)					
0.72	36.45	27.22	1.35	1,283.08	99.51
0.82	36.28	26.96	1.36	1,283.03	99.17
SEM	0.50	0.43	0.02	0.19	0.29
SSM (%)					
0	37.59^a^	25.97^b^	1.45^a^	1,283.25	100.48^a^
10	36.15^ab^	27.13^ab^	1.35^ab^	1,283.10	99.42^ab^
20	35.35^b^	28.18^a^	1.26^b^	1,282.82	98.10^b^
SEM	0.61	0.53	0.03	0.24	0.36
Phy (g/ton)					
0	36.44	27.13	1.36	1,283.13	99.66
300	36.29	27.06	1.35	1,282.99	99.00
SEM	0.50	0.43	0.02	0.19	0.29
Lys×SSM×Phy					
0.72×0×0	37.34	26.18	1.43	1,283.39	100.57
0.72×10×0	36.59	27.35	1.34	1,283.19	99.71
0.72×20×0	35.35	28.14	1.26	1,283.00	98.17
0.82×0×0	37.69	26.01	1.46	1,283.24	100.96
0.82×10×0	36.19	26.88	1.37	1,283.09	99.87
0.82×20×0	35.47	28.19	1.27	1,282.85	98.68
0.72×0×300	37.85	26.01	1.46	1,283.11	100.23
0.72×10×300	36.14	27.43	1.34	1,283.02	98.94
0.72×20×300	35.43	28.22	1.25	1,282.78	98.12
0.82×0×300	37.48	25.69	1.47	1,283.24	100.16
0.82×10×300	35.68	26.87	1.34	1,283.10	99.17
0.82×20×300	35.16	28.16	1.25	1,282.66	98.17
SEM	1.23	1.07	0.06	0.48	0.72
p-value					
Lys	0.81	0.67	0.76	0.86	0.44
SSM	0.03	0. 01	0.0005	0.46	0.0001
Phy	0.83	0.91	0.91	0.61	0.12
Lys×SSM	0.96	0.94	0.98	0.97	0.88
Lys×Phy	0.78	0.92	0.78	0.76	0.93
SSM×Phy	0.93	0.97	0.87	0.98	0.98
Lys×SSM×Phy	0.98	0.99	0.99	0.98	0.94

LDL, low-density lipoprotein; HDL, high-density lipoprotein; SEM, standard error of the mean; SSM, sesame seed meal.

a,b Means within a column with same or no superscript do not differ significantly (p>0.05).

**Table 7:** Effect of experimental diets on serum glutathione peroxidase (GSH-Px) activity, malondialdehyde (MDA), total superoxide dismutase (TSOD) and total antioxidant capacity (T-AOC)

Items	GSH-Px (U/mL)	MDA (nmol/mL)	TSOD (U/mL)	T-AOC (U/mL)
Lys (%)				
0.72	820.29	5.04	150.79	6.25
0.82	820.53	5.04	151.25	6.26
SEM	0.85	0.08	0.89	0.02
SSM (%)				
0	818.49	5.17	148.72^b^	6.19^b^
10	821.15	4.99	151.28^ab^	6.26^ab^
20	821.59	4.95	153.06^a^	6.32^a^
SEM	1.04	0.10	1.09	0.03
Phy (g/ton)				
0	820.26	5.05	150.89	6.25
300	820.56	5.02	151.15	6.26
SEM	0.85	0.08	0.89	0.02
Lys×SSM×Phy				
0.72×0×0	818.34	5.19	148.74	6.21
0.72×10×0	820.71	4.99	150.36	6.29
0.72×20×0	820.99	4.92	152.57	6.33
0.82×0×0	818.34	5.18	148.25	6.18
0.82×10×0	821.15	5.04	152.07	6.22
0.82×20×0	821.64	4.98	153.34	6.26
0.72×0×300	818.30	5.18	148.90	6.19
0.72×10×300	821.56	4.99	151.24	6.20
0.72×20×300	821.86	4.95	152.96	6.27
0.82×0×300	818.99	5.15	148.98	6.20
0.82×10×300	820.81	4.93	151.46	6.32
0.82×20×300	821.86	4.95	153.39	6.40
SEM	2.09	0.21	2.18	0.06
p-value				
Lys	0.84	0.99	0.71	0.73
SSM	0.08	0.30	0.02	0.03
Phy	0.79	0.83	0.83	0.67
Lys×SSM	0.99	0.98	0.92	0.89
Lys×Phy	0.83	0.81	0.86	0.07
SSM×Phy	0.98	0.98	0.99	0.90
Lys×SSM×Phy	0.92	0.98	0.94	0.59

SEM, standard error of the mean; SSM, sesame seed meal.

a,b Means within a column with same or no superscript do not differ significantly (p>0.05).
